# Anemia, Iron Deficiency, and Iron Regulators in Pancreatic Ductal Adenocarcinoma Patients: A Comprehensive Analysis

**DOI:** 10.3390/curroncol30080560

**Published:** 2023-08-18

**Authors:** Malgorzata Osmola, Beata Gierej, Katarzyna Mleczko-Sanecka, Aneta Jończy, Olga Ciepiela, Leszek Kraj, Bogna Ziarkiewicz-Wróblewska, Grzegorz Władysław Basak

**Affiliations:** 1 Department of Hematology, Transplantation, and Internal Medicine, University Clinical Centre, Medical University of Warsaw, 02-097 Warsaw, Poland; 2 Department of Pathology, University Clinical Centre, Medical University of Warsaw, 02-097 Warsaw, Poland; hematologia@wum.edu.pl (B.G.);; 3 Department of Pathology and Laboratory Medicine, Maria Skłodowska-Curie National Oncology Research Institute, 02-781 Warsaw, Poland; 4 International Institute of Molecular and Cell Biology, 02-109 Warsaw, Poland; 5 Department of Laboratory Medicine, Medical University of Warsaw, 02-097 Warsaw, Poland; 6 Department of Oncology, University Clinical Centre, Medical University of Warsaw, 02-097 Warsaw, Poland

**Keywords:** iron deficiency, iron metabolism, anemia, pancreatic cancer, vitamin B12 deficiency, hepcidin, ferroportin, ZIP14

## Abstract

Anemia and iron deficiency (ID) are common complications in patients with pancreatic ductal adenocarcinoma (PDAC), but their underlying causes remain unclear. This study investigated the incidence and characteristics of anemia and micronutrient deficiencies in PDAC patients before initiating chemotherapy. A total of 103 PDAC patients were included, comprising 67 in the palliative and 36 in the adjuvant groups. The overall incidence of anemia was 42.7% (*n* = 44), with comparable rates in both groups. Normocytic and normochromic anemia were predominant, with mild and moderate cases observed in 32% and 10.7% of the cohort, respectively. ID was evident in 51.4% of patients, with absolute ID more frequent in the adjuvant than in the palliative group (19.4% vs. 13.4%). Functional ID occurred more often in the palliative than in the adjuvant group (41.8% vs. 25%). Vitamin B12 and folate deficiency occurred in <5% (*n* = 5) of patients. Furthermore, 8.7% (*n* = 9) of patients had chronic kidney disease and anemia. To elucidate mechanisms of iron deficiency, the study explored the expression of iron regulators (hepcidin (HEP), ferroportin (FPN), and ZIP14 protein) and mitochondrial mass in PDAC tissue with immunohistochemical (IHC) staining and Perl’s Prussian blue to detect iron deposits on available tumor samples (*n* = 56). ZIP14 expression was significantly higher in less advanced tumors (*p* = 0.01) and correlated with mitochondrial mass (*p* < 0.001), potentially indicating its role in local iron homeostasis. However, no significant impact of tissue iron regulators on patient survival was observed. Perl’s Prussian blue staining revealed iron deposits within macrophages, but not in pancreatic duct cells. Furthermore, the GEPIA database was used to compare mRNA expression of iron regulators (HEP, FPN, and ZIP14) and other genes encoding iron transport and storage, including Transferrin Receptor Protein 1 (TfR1) and both ferritin chain subunits (FTH and FTL), in PDAC and normal pancreatic samples. FPN, TfR1, FTH, and FTL showed higher expression in tumor tissues, indicating increased iron usage by cancer. ZIP14 expression was higher in the pancreas than in PDAC and was correlated with FPN expression. The study highlights the importance of baseline iron status assessment in managing PDAC patients due to the high incidence of anemia and iron deficiency. Furthermore, ZIP14, in addition to HEP and FPN, may play a crucial role in local iron homeostasis in PDAC patients, providing valuable insights into the underlying mechanisms of iron dysregulation.

## 1. Introduction

Anemia is frequent in cancer patients, affecting approximately one in two individuals undergoing systemic treatment and one in three before therapy initiation [1,2]. Among the various types of anemia in cancer, iron deficiency (ID) and chronic disease anemia are the most prevalent, which often overlap and are aggravated by blood loss, micronutrient deficiencies, and chronic kidney disease [3,4,5]. Anemia and ID result in physical symptoms, including decreased exercise capacity, dizziness, and decreased quality of life [4,6], whereas iron repletion improves patients’ physical performance [7].

Patients with pancreatic ductal adenocarcinoma (PDAC) face an exceptionally high risk of anemia [2,8,9,10]. While the underlying mechanisms responsible for anemia in PDAC are not fully understood, ID has been observed in over 60% of these patients [2]. Iron plays a vital role in multiple cellular functions [11,12], and cancer cells, with their high metabolic activity and rapid proliferation rates, rely heavily on iron for growth and survival [13]. Consequently, the increased iron utilization by cancer cells leads to systemic ID. In PDAC, elevated iron usage is attributed to mitochondrial metabolism, where iron is an essential component of the respiratory chain, enabling ATP production and reactive oxygen species generation [14,15]. 

Moreover, malignancy-associated inflammation contributes to the elevated production of hepcidin (HEP), a key iron regulator, leading to the degradation and blockage of the iron exporter ferroportin (FPN). This, in turn, reduces iron absorption in the digestive tract and promotes iron retention within macrophages [16,17,18]. Another critical player in iron regulation is Zrt- and Irt-like protein (ZIP) 14, which functions as a cellular iron importer, especially for non-transferrin-bound iron (NTBI) and other ions [19]. Notably, emerging evidence underscores the significance of the tumor microenvironment, specifically the presence of macrophages, in modulating iron homeostasis within the tumor [20].

In this study, we aimed to investigate the prevalence of anemia in patients diagnosed with PDAC at the time of inclusion for chemotherapy, whether adjuvant or palliative. Additionally, we sought to analyze potential causes of anemia, including micronutrient deficiencies (such as iron, vitamin B12, and folate) and chronic kidney disease, the most common underlying factor in anemia associated with chronic disease. To shed light on the possible mechanisms of iron deficiency in PDAC, we performed immunohistochemical (IHC) staining to examine the expression levels of key iron regulators—HEP, FPN, and ZIP14—within the cancer tissue. Furthermore, we assessed mitochondrial mass as an indicator of iron-dependent cellular metabolic requirements. We performed Perl’s Prussian blue staining to determine whether PDAC cells and surrounding pancreatic ducts contain iron deposits. We performed an analysis using the GEPIA database to compare the mRNA expression of HEP, FPN, ZIP14, and other genes encoding iron transport and storage Transferrin Receptor Protein 1 (TfR1) as well as both ferritin chain subunits (FTH and FTL) in PDAC and normal pancreatic samples. Finally, we explored potential correlations among the measured variables, providing a comprehensive understanding of the complex interplay between iron metabolism and anemia in PDAC patients.

## 2. Materials and Methods

We performed a retrospective, observational analysis of 103 patients diagnosed with PDAC before the onset of chemotherapy, treated between 11.2012 and 9.2020 at the Department of Hematology, Oncology, and Internal Medicine at the Central University Hospital, Medical University of Warsaw, Poland. The inclusion criteria were histologically confirmed PDAC diagnosis, chemotherapy administration in a palliative (treatment given in the non-curative intention) or adjuvant (following tumor’s resection) setting, no chemotherapy given before the blood sample, and tissue sample analysis. We performed IHC analysis on archival tissue samples from the primary tumor of 56 out of 103 patients initially included in the study. PDAC samples from metastases or insufficient tissue samples to perform IHC staining were the two factors limiting the performance of IHC studies in every patient. Overall survival was defined as the time from the start of chemotherapy to the day of death; these data were retrieved from the Polish National Health Fund.

### 2.1. Blood Analysis

Data from blood laboratory assessments carried out according to the local protocol were retrieved from patients’ past medical histories. The analysis included a complete blood count; iron-related, micronutrient, and renal function parameters were assessed 0–14 days before the onset of chemotherapy and 14–90 days after the surgery or diagnostic biopsy. Serum iron and micronutrient concentrations were available for 96 out of 103 patients in the cohort. Chronic kidney disease (CKD) was defined as the glomerular filtration rate (GFR) < 60 mL/min/m^2^ measured using the Cockcroft–Gault Formula. At the time of analysis, no one from our cohort was under iron, vitamin B12, or folate supplementation according to data from the past medical history. We applied standard definitions of ID and anemia (hemoglobin level Hb < 12 g/dL as the anemia cut-off was used [21,22] and was further classified as mild, Hb 10.0–11.9 g/dL; moderate, 8.0–9.9 g/dL; and severe, <8.0 g/dL). ID was defined as transferrin saturation (TSAT) below 20% and was further divided into absolute ID (TSAT < 20% and serum ferritin (SF) < 100 ng/mL) or functional ID (TSAT < 20% and SF ≥ 100 ng/mL) [4,22]. Vitamin B12 and folate were assessed by electrochemiluminescence (ECLIA) using a Roche Cobas e 801 analyzer. Serum ferritin and transferrin were assessed by an immunoturbidimetric assay, whereas iron and creatinine concentrations were with a colorimetric test on Roche Cobas c 702 analyzer. In complete blood count, fluorescence flow cytometry was used with the SLS method for hemoglobin detection on Sysmex XN 2000 analyzer. TIBC and TSAT are derivatives of serum iron and transferrin. Table 1 contains the study definitions.

### 2.2. Immunohistochemical and Histological Staining in Tissue Samples

PDAC tissue samples were obtained from curative or diagnostic surgery or core biopsies. The samples were fixed in 10% formaldehyde and embedded in paraffin. The pathological stage was diagnosed according to the Union for International Cancer Control TNM Classification of Malignant Tumors, 8th edition [23]. The tumor’s TNM, grade, histopathological subtype, location, vascular and neural invasion, and resection were assessed where applicable.

Paraffin blocks were cut into 4 μm sections and placed on the glass slides. Next, the sections were deparaffinized and treated with an antigen retrieval solution pH 9.0 using a DAKO PT Link device. Endogenous peroxidase activity was blocked using 3% hydrogen peroxide for 5 min, and the sections were incubated for 30 min at room temperature with primary antibodies (anti-hepcidin-25 antibody: rabbit polyclonal, dilution 1:50, Abcam, product code: ab30760; anti-ferroportin: anti-SLC40A1 antibody, rabbit polyclonal, dilution 1:1000, Abcam, product code: ab78066; anti-ZIP-14: anti-SLC39A14 antibody, rabbit polyclonal, dilution 1:2500, Sigma-Aldrich, product code: HPA016508; and anti-mitochondria: 113-1 antibody, mouse monoclonal, dilution 0.5:1000, Novus Biologicals, product code: NBP2-32980-0.2 mg). Afterward, sections were treated with a linker for 15 min at room temperature (apart from the anti-ZIP14 antibody). As positive controls, we used liver tissue for HEP, colon for FPN, small intestine for ZIP14, and oncocytoma for mitochondrial mass. To visualize the staining intensity, we used EnVision FLEX. All sections were then counterstained with hematoxylin. We performed the procedures according to the manufacturer’s instructions. IHC staining was evaluated by two experienced pathologists blinded for clinical data (B. G. and B.Z.W).

The staining intensity of HEP, FPN, ZIP, and mitochondrial mass was evaluated in relation to the surrounding pancreatic duct cells (endogenous control). Stained sections were classified into three categories: “0” as negative (without expression); “1” as weakly stained (stained weaker than control); and “2” as strongly stained (stained equal to or stronger than control). The expression of mitochondrial mass was classified into two categories: “1” as weakly stained (stained weaker than surrounding pancreatic ducts) and “2” as strongly stained (stained equal or stronger than surrounding pancreatic ducts). The negative staining of mitochondrial mass was not applicable due to the physiological presence of mitochondria within the cells, translating into the presence of IHC expression. To perform correlations in the statistical analysis, we divided IHC intensity into two groups: not stained and stained group (including both weakly and strongly stained).

We performed Perl’s Prussian blue staining to determine whether PDAC cells and the surrounding pancreatic ducts contain iron deposits. For this purpose, we used the Bio-Optica Milano S.p.A. set (Code: 04-180807) kit. Sections were immersed for 20 min in 10 mL of reagent A, containing a potassium ferrocyanide solution; 30 mL of distilled water; and 4 mL of reagent B, containing acid activation buffer. Subsequently, sections were washed in distilled water and 10 drops of reagent C with carmalum, according to Mayer, and left for 5 min. Finally, sections were washed with distilled water, dehydrated in ascending alcohols, cleared in xylene, and mounted. We performed the procedure according to the manufacturer’s instructions. As a positive control, a liver sample with hemochromatosis was used.

### 2.3. GEPIA Database Analysis

GEPIA (gene expression profiling interactive analysis) was used to compare the mRNA expression of HEP (Hamp), FPN (Slc40a1), ZIP14 (Slc39a14), and other genes encoding iron transport and storage, including Transferrin Receptor Protein 1 (TfR1) and both ferritin chain subunits (FTH and FTL), in PDAC and normal pancreatic samples [24]. To visualize the data, the following parameters were applied within the analysis: |Log2FC|, cutoff:1, and *p*-value cutoff at 0.01.

### 2.4. Statistical Analysis

The values of the quantitative variables analyzed are presented as means and standard deviation, and qualitative variables by number and percentage. The Chi-square test was used to assess the relationship of the qualitative variables analyzed. To check for the normal distribution of quantitative variables, the Shapiro–Wilk test was performed. To test the differences between the two groups, the Student’s *t*-test was used, and if the conditions for its application were not met, the Mann-U-Whitney test was used. To assess the relationship between some of the variables, the rank correlation coefficient was used (Spearman’s rank correlation coefficient). The Kaplan–Meier method evaluated the survival rate, and the log rank tested the differences between survival curves. The level of significance, a *p*-value of 0.05, was adopted. Cox analysis was performed for multivariate analysis. Database and statistical tests were performed using Statistica 9.1 software (StatSoft, Poland).

## 3. Results

### 3.1. Anemia and Its Possible Causes in Patients with PDAC before the Onset of Chemotherapy

Our study measured the incidence of anemia, iron, and other micronutrient deficiencies in 103 patients with PDAC at the time of inclusion to chemotherapy. The cohort was further divided into the palliative (*n* = 67) and adjuvant (*n* = 36) groups since the causes of anemia in those patients might differ (e.g., excessive blood loss during surgery in patients undergoing tumor resection in the adjuvant group). Clinical data, serum-iron-related, and micronutrient variables in PDAC patients are presented in Table 1.

#### 3.1.1. Anemia and Its Characteristics

We detected anemia in 42.7% of patients with PDAC, with similar results in the adjuvant and palliative group (41.6% and 43.2%, respectively), and mild and moderate anemia occurring in 32% and 10.7% in the whole cohort, respectively. Anemia was normocytic (MCV 80–100 fL) and normochromic (MCHC > 31 g/dL) in most cases (91.3% and 98.2%, respectively), whereas microcytic and macrocytic anemia occurred in 6.7% and 1.8% in the whole cohort, respectively (Table 1).

#### 3.1.2. Iron Deficiency

**Figure 1:**
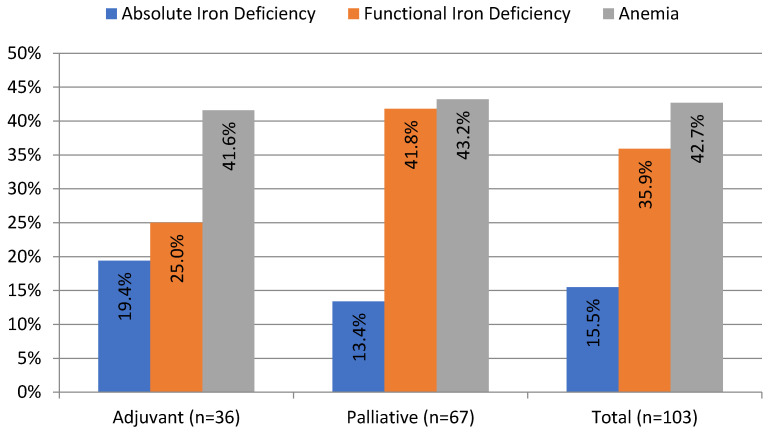
Prevalence of anemia and iron deficiency in patients with pancreatic ductal adenocarcinoma before the onset of chemotherapy. Anemia (Hb < 12 g/dL); absolute iron deficiency (transferrin saturation (TSAT) < 20%; serum ferritin (SF) < 100 ng/mL); and functional iron deficiency (TSAT < 20; SF ≥ 100 ng/mL).

ID, defined as TSAT < 20%, was present in 51.4% of patients in our cohort. Absolute ID (TSAT < 20% and SF < 100 ng/mL) occurred more often in the adjuvant than in the palliative group (19.4% vs. 13.4%, although the difference was not statistically significant). Half of the patients with absolute ID (*n* = 8 out of 16) presented with anemia. Functional ID (TSAT < 20% and SF ≥ 100 ng/mL) occurred twice more often than absolute ID and was present in 35.9% of patients; it occurred more often in the palliative than in the adjuvant group, although not statistically significant (41.8% vs. 25%, *p* = 0.1). Overall, 62% of patients with functional ID (*n* = 23 out of 37) had anemia (Table 1). The prevalence and different types of ID are presented in Figure 1.

#### 3.1.3. Vitamin B12 and Folate Deficiency

**Figure 2:**
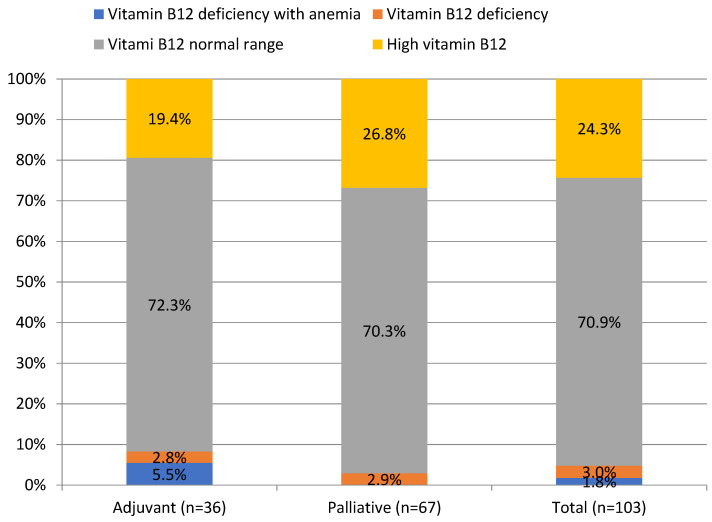
Prevalence of vitamin B12 deficiency in patients with pancreatic ductal adenocarcinoma.

Vitamin B12 and folate deficiency anemia occurred in 1.8% and 2.9% of patients, respectively, whereas their deficiency, regardless of anemia, was found in 4.8% and 7.7% of patients, as shown in Table 1. Mean folate concentration was significantly lower in the palliative than in the adjuvant group (7.3 ± 3.2 vs. 9.4 ± 5.1 ng/mL, *p* = 0.01), as shown in Table 1. Interestingly, elevated vitamin B12 levels were noted in 24.3% of PDAC patients, as shown in Figure 2. 

#### 3.1.4. Chronic Kidney Disease

Since anemia can be attributed to concurrent kidney dysfunction due to declined erythropoietin production, we searched for correlations between kidney function, red blood cell parameters, and iron-related variables. In our cohort, 15.5% (*n* = 16) of patients showed CKD, with a mean creatinine concentration of 0.7 ± 0.2 mg/dL in the whole cohort, with 8.7% (*n* = 9) of patients having CKD and anemia, as shown in Table 1. Mean Hb and Ht concentrations in patients with CKD were lower than in patients with preserved kidney function (11.5 ± 1.2 g/dL and 35.1 ± 3.5% vs. 12.2 ± 1.6 g/dL and 36.0 ± 4.3%, although the differences were not statistically significant at *p* = 0.07). Mean MCV was significantly higher in patients with CKD than in patients with preserved kidney function (93.6 ± 5.3 vs. 89.0 ± 4.9 fL, *p* = 0.002), although it was within the normal range. There were no differences regarding serum-iron-related variables between patients with chronic kidney disease and preserved kidney function. Differences in patients with and without chronic kidney regarding red blood cell parameters and iron status are presented in Table 2.

### 3.2. Immunohistochemical and Histochemical Study Results

**Figure 3:**
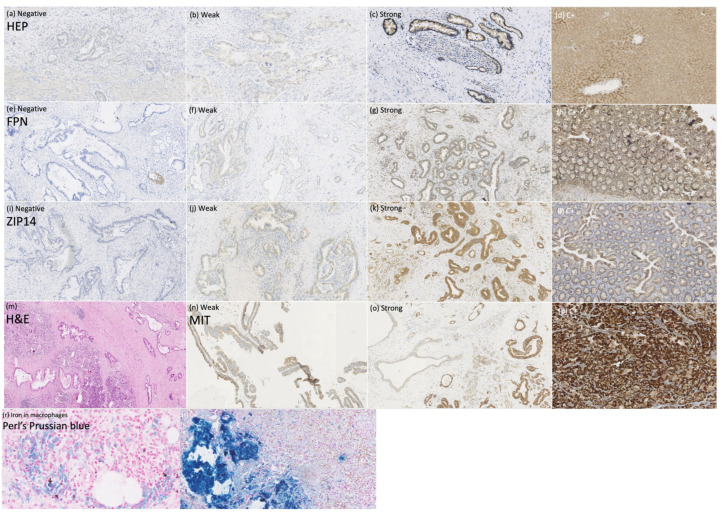
Immunohistochemical and histochemical studies on pancreatic ductal adenocarcinoma tissue. Anti-hepcidin (HEP) staining (**a**–**d**), magnification × 100: (**a**) negative, (**b**) weak, (**c**) strong, (**d**) positive control; anti-ferroportin (FPN) staining (**e**–**h**), magnification × 100: (**e**) negative, (**f**) weak, (**g**) strong, (**h**) positive control; anti-ZIP14 staining (**i**–**l**), magnification × 100: (**i**) negative, (**j**) weak, (**k**) strong, (**l**) positive control; (**m**) pancreas with ductal adenocarcinoma infiltration, hematoxylin-eosin staining, magnification × 50; anti-mitochondrial (MIT) staining, (**n**) weak, (**o**) strong, (**p**) positive control; (**r**) Perl’s Prussian blue staining of the macrophages, magnification × 400; (**s**) positive control, magnification × 200.

To explain the mechanisms of iron deficiency in patients with PDAC, we assessed the expression of the iron regulators HEP, FPN, and ZIP14 in PDAC tissue (*n* = 56) to determine whether they can be expressed locally by the tumor and influence the tumor’s iron homeostasis, as well as the mitochondrial mass to search for the link between iron regulators and mitochondrial metabolism within PDAC. Additionally, we performed Perl’s Prussian blue staining to look for iron deposits within the PDAC cells. The staining of HEP, FPN, ZIP14, MIT, and Perl’s Prussian blue in PDAC tissue are presented in Figure 3.

HEP and FPN staining were negative in 28.6% and 60.7%, weak in 42.9% and 25%, strong in 28.6% and 14.3%, respectively, in the whole cohort. No significant differences were noted between the adjuvant and palliative groups in HEP and FPN expression. Still, a percentage of negative FPN expression in the whole cohort and in the palliative group of 60.7% (*n* = 34) and 71.4% (*n* = 15), respectively, is worth underlining. HEP tended to correlate negatively with FPN expression, but this correlation did not reach statistical significance (Chi-Square test, *p* = 0.051; R-0.25 Spearman’s correlation).

**Figure 4:**
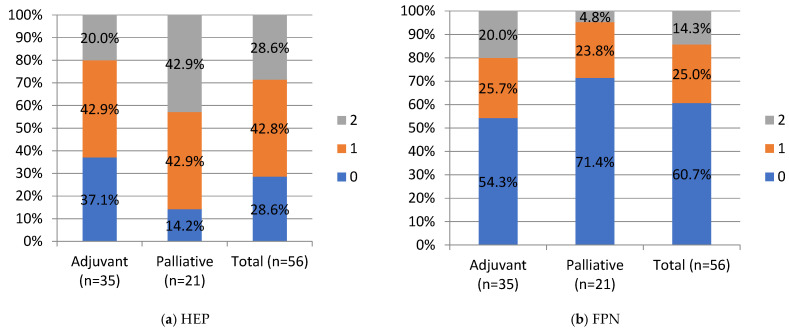

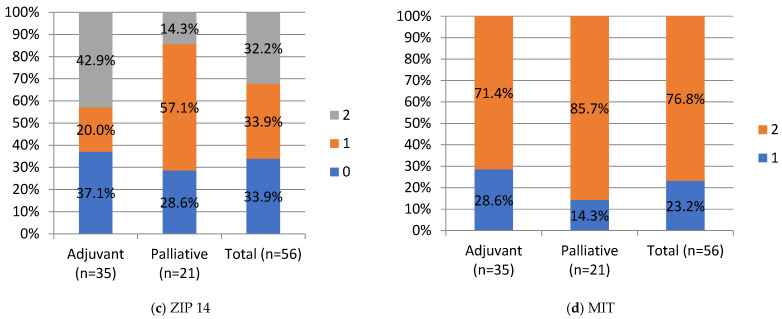
IHC staining intensity in patients with PDAC. HEP, hepcidin; FPN, ferroportin; ZIP, Zrt- and Irt-like protein transporter 14; MIT, mitochondrial mass. Staining intensity, 0, 1, 2: “0” (negative) for lesions without expression; “1” (weakly stained) for lesions stained weaker than control; “2” (strongly stained) for lesions stained equal to or stronger than control. For ZIP14, there was a statistical difference between the adjuvant and palliative groups, *p*-value = 0.01 (Chi-Square test). Values are presented as percentages of results.

Negative and moderate ZIP14 staining was noted in 37.1% (*n* = 13) and 20% (*n* = 7), in the adjuvant group and 28.6% (*n* = 6) and 57.1% (12) in the palliative group, respectively. Strong staining was noted in 42.9% (*n* = 15) of the adjuvant and 14.3% (*n* = 3) of the palliative group. A significant difference was noted in ZIP14 expression between the adjuvant and palliative groups (*p* = 0.01, Chi-Square test). Regarding correlations, ZIP14 correlated with mitochondrial mass (MIT) (*p* < 0.001, Chi-square test). Low MIT content was observed in patients with high ZIP14 expression (i.e., more mitochondria are present in PDAC with stronger ZIP14 expression). Low MIT content was noted in 23% (*n* = 13) of PDAC patients, and the majority, 76.8% (*n* = 43), showed high MIT content in the whole group. The differences in IHC expressions between the adjuvant and palliative groups are presented in Figure 4.

Perl’s Prussian blue stating revealed iron deposits only within the macrophages and not in the pancreatic duct’s cells (Figure 3r).

### 3.3. Survival Analysis

**Figure 5:**
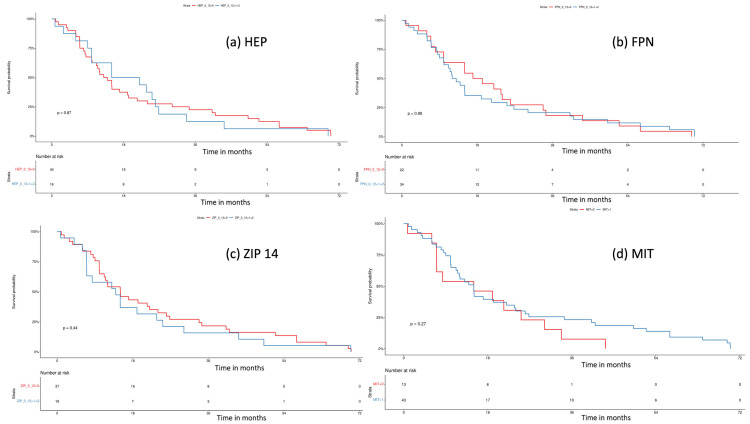
Survival analysis with log-rank test for (**a**) hepcidin (HEP), “0” (negative) vs. “1” + ”2”, (weak and strong expression), *p* = 0.8; (**b**) ferroportin (FPN), “0” (negative) vs. “1” + ”2” (weak and strong expression), *p* = 0.8; (**c**) ZIP 14, “0” (negative) vs. “1” + ”2” (weak and strong expression), *p* = 0.4; (**d**) mitochondrial mass (MIT), “0” (weak) vs. “1” (strong expression), *p* = 0.3.

We performed a log-rank test to assess the influence of tissue iron regulators on the patient’s survival. Median overall survival (OS) was “0” vs. “1” and “2” (13.5 months, 95% CI 10.1–21.4 vs. 18.5 months, 95% CI 10–33.77; *p* = 0.87) for HEP; “0” vs. “1” + “2” (18.1 mo, 95% CI 10–33.8 vs. 12.5 mo, 95% CI 10.1–21.4; *p* = 0.88) for FPN; “0” vs. “1” + “2” (15.0 mo, 95% CI 11.2–26 vs. 13.9 95% CI 7.0, 30.2; *p* = 0.44) for ZIP14; and “0” vs. “1” (15 mo, 95% CI 7–NA vs. 15 mo, 95% CI 11.2–24; *p* = 0.27) for MIT. Overall, we did not notice any impact of tissue iron regulators on the patient’s survival. Kaplan–Meier curves are presented in Figure 5.

### 3.4. GEPIA Database Analysis

**Figure 6:**
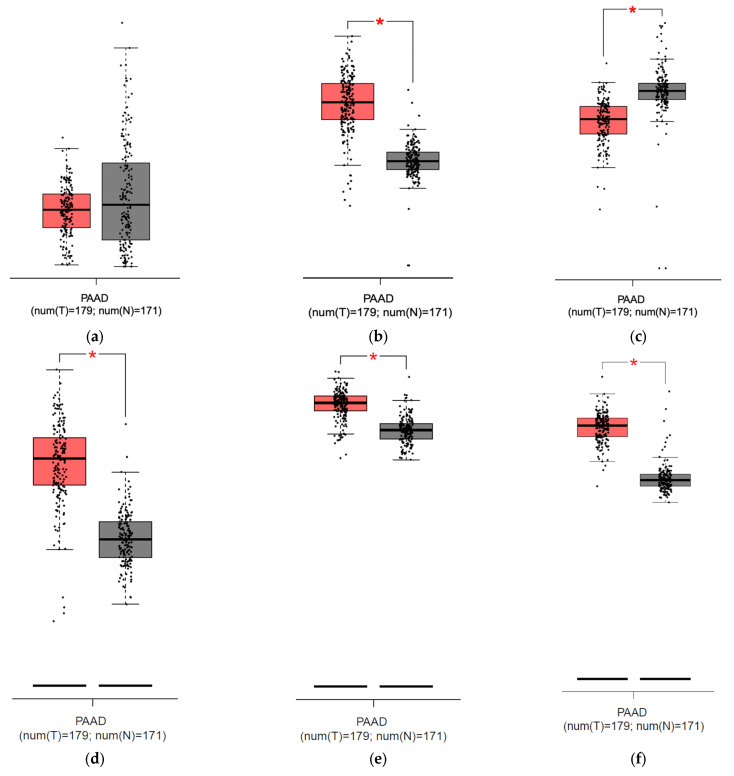

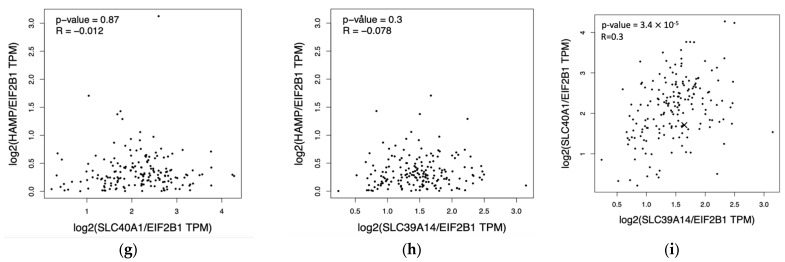
Results from analysis from the GEPIA database for pancreatic adenocarcinoma (PAAD): (**a**) hepcidin expression in the pancreas (grey) and pancreatic cancer (red), no significant difference detected; (**b**) ferroportin expression is significantly higher [*] in pancreatic cancer (red) than in pancreas (grey); (**c**) ZIP14 expression is significantly lower [*] in pancreatic cancer (red) than in pancreas (grey); (**d**) Transferrin Receptor Protein 1 (TfR1) expression is significantly higher [*] in pancreatic cancer (red) than in pancreas; (**e**) ferritin heavy chain (FTH) expression is significantly higher [*] in pancreatic cancer (red) than in pancreas (grey); (**f**) ferritin light chain (FTL) expression is significantly higher [*] in pancreatic cancer (red) than in pancreas (grey); (**g**) correlation between mRNA expression of HEP (HAMP) and FPN (SLC40A1), *p* = 0.87; (**h**) correlation between mRNA expression of HEP (HAMP) and ZIP14 (SLC39A14), *p* = 0.3; (**i**) correlation between FPN (SLC40A1) and ZIP14 (SLC39A14), R = 0.3, *p* < 0.001 [24].

To compare the mRNA expression between PDAC and normal pancreatic samples of HEP, FPN, ZIP14, and additional iron transport and storage proteins TfR1 and ferritin chain subunits (FTH and FTL) to find correlations confirming a high iron usage in PDAC, we employed the GEPIA database. The analysis showed no significant differences between HEP expression between the normal pancreas and pancreatic cancer but revealed a higher expression of FPN in the tumor than in the normal pancreas; there was also no correlation between HEP and FPN nor between HEP and ZIP14 (Figure 6a,b,g,h). Consistently, our analysis showed stronger ZIP14 expression in the pancreas compared to PDAC and existing correlations between FPN and ZIP14 expression (Figure 6c,i). Additionally, our analysis showed higher TfR1, FTH, and FTL expressions within the PDAC cells than in the normal pancreatic samples (Figure 6d–f).

## 4. Discussion

Our study revealed that 42.7% of patients with PDAC were anemic before the onset of systemic treatment. Anemia occurred more often in PDAC patients than in the cancer patients reported in a study by Ludwig et al. (33%) [2]. Importantly, we found that anemia was normocytic in most cases, with microcytic anemia noted only in 6.7% of patients. In clinical practice, microcytosis is often considered a symptom of ID [25]. Hence, our observations imply that this approach seems inadequate in cancer patients.

We detected ID in 51.4% of study participants. Absolute ID, which reflects depleted iron stores, was more frequent in the adjuvant group, although not significantly. It might be explained by undergoing extensive surgery with excessive blood loss. Pancreatoduodenectomy, a surgical procedure most often performed due to PDAC, is associated with a high prevalence of anemia and ID in follow-ups [8,9,10], which may be caused by occult bleeding after the procedure and malabsorption of the micronutrients from the gastrointestinal tract [4]. Functional ID, in which inefficient utilization of iron stores is observed, occurred more frequently than absolute ID and was more frequent in the palliative than the adjuvant group (although not statistically significant). Patients treated palliatively have more advanced disease and a more pronounced inflammation process that leads to functional ID [26,27]. 

Vitamin B12 and folate deficiency anemia in PDAC patients initiating systemic therapy with adjuvant or palliative intent was assessed for the first time. We found that vitamin B12 and folate deficiency were rare findings. Studies indicate that only 2% of patients are diagnosed with vitamin B12 deficiency after pancreatoduodenectomy, and none show folate deficiency [8]. Interestingly, high vitamin B12 was noted in one-fifth of PDAC patients in our cohort. The cause of elevated vitamin B12 remains unknown, but it is described as a poor prognostic factor in patients with advanced cancer [28,29]. Folate concentration was significantly lower in the palliative than in the adjuvant group but remained within the normal range and should not have clinical implications. Although guidelines for anemia management in cancer patients (e.g., ESMO) suggest the assessment of vitamin B12 and folate concentration, the prevalence of their deficiencies was not studied before on large groups of cancer patients, including PDAC.

CKD can contribute to the development of anemia through decreased erythropoietin synthesis and increased hepcidin levels, leading to iron sequestrations and functional ID, including in cancer patients [5,30,31]. Our study revealed that PDAC patients with CKD were not more often anemic than patients with preserved kidney function, and iron-related parameters were similar in both groups. Of note, the mean Hb level was lower in the CKD group, although it was not significantly different, indicating that CKD might be an essential factor in developing anemia in cancer patients. However, studies on bigger groups are needed to assess the role of kidney function on anemia in cancer patients. 

HEP in malignancy is elevated due to increased liver synthesis and local tumor production [32,33,34,35]. An important question is whether dysregulated HEP–FPN axis contributes to PDAC growth through altered tumor iron homeostasis. In our study, HEP expression in cancer tissue tended to correlate with FPN in the whole cohort, suggesting a minor contribution of local hepcidin production to FPN downregulation. Our results implied that FPN expression might be predominantly controlled by systemic HEP production or that there is an alternative FPN regulation in PDAC. In our study, FPN expression was mostly negative, but it did not affect the overall survival in patients (Figure 5b). Still, some studies show that low FPN expression in pancreatic and breast cancer is a negative predictive factor for survival [33,36].

Our study revealed a statistical difference in IHC staining intensity for ZIP14 between the adjuvant and the palliative group, with a higher expression in the adjuvant group translating into lower expression in more advanced tumors. The same observations come from the study on prostate cancer, revealing lower expression in more advanced prostate cancer and a higher risk of disease recurrence [37]. Our analysis in the GEPIA database consistently showed higher ZIP14 expression in the pancreas compared to pancreatic cancer (Figure 6c). A similar observation was made for hepatic tissue and hepatocellular carcinoma [38]. In contrast, another study showed that a higher expression of ZIP transporters (excluding ZIP14) on mRNA level predicts a worse prognosis in patients with PDAC [38]. ZIP14 is an NTBI transporter, but in our study, we detected iron deficiency rather than iron overload, a condition that typically leads to the systemic presence of NTBI. However, iron in the form of NTBI may be absorbed locally from the tumor’s microenvironment [35]. Additionally, ZIP14 delivers other ions (e.g., manganese and zinc). Another study shows the induction of ZIP14 and altered zinc homeostasis in muscles with PDAC-associated cachexia in murine models [39]. In our study, ZIP14 expression correlated with a mitochondrial mass within the PDAC cells, concluding that ZIP14 potentially delivers ions for mitochondria that might support carcinogenesis. Another study revealed that ZIP14 provides ions for intracellular metabolism (both mitochondrial and glycolysis in the cytosol), and ZIP14 knockout mice had lower cytosolic ATP in the liver. Impaired glycolysis in mice was restored by zinc supplementation [40,41]. The correlation of ZIP14 in PDAC with mitochondrial mass might indicate that ZIP14 is responsible for delivering ions (not only iron but others, like zinc) for metabolism within the PDAC.

ZIP14 might be a biomarker for hepatocellular carcinoma and colorectal cancer due to different splicing of ZIP14 in the cancer tissue compared to healthy tissue [41]. Still, studies of this phenomenon in PDAC have not been performed so far, but our study adds an important insight into ZIP14′s presence in this cancer. Nevertheless, further studies are necessary to reveal the clinical implications of those findings. 

Additionally, we analyzed TfR1, ferritin chains (FTH and FTL), in the GEPIA database to search for other markers that would indicate high iron usage within PDAC cells. Analyses showed higher expression of those markers in pancreatic cancer than in normal pancreas, indicating a high iron usage by PDAC. TfR1 is a receptor that mediates iron release into the cytoplasm after endocytosis, and ferritin is an iron-storage protein complex composed of two chains: light and heavy. TfR1, FTH, and FTL upregulation has been confirmed in tumorigenesis [42,43,44]

Since we noticed significant changes in iron-related proteins, we performed Perl’s staining to detect the presence of iron in tissue samples. We would expect iron in the cells in case of high HEP and low FPN expression. Nevertheless, Perl’s staining revealed no iron deposits within PDAC cells, implying that iron must be actively used. The only cells that were stained for iron’s presence were macrophages. The relevance of tumor-associated macrophages in PDAC is emerging, and recent studies reveal its role in tumor progression; nevertheless, its association with iron transport within PDAC is unknown [43,44].

Our study has several limitations. First, small study groups made formulating any conclusions difficult, especially from the IHC staining. Second, we did not assess systemic hepcidin concentration; only IHC staining on PDAC tissue was performed, which is a critical drawback since hepcidin production occurs mainly in the liver. Additionally, other parameters for impaired iron status (e.g., percentage of hypochromic cells, hemoglobin content of reticulocytes, and the soluble transferrin receptor) were not assessed. The tissue and serum samples were collected on different days, and the latter were collected at the inclusion for chemotherapy. Therefore, we could not conclude the correlation between the iron regulator’s expression on an autocrine level and compare them with iron-related variables in the serum. We could not perform IHC analysis on the tissue samples in all patients (only for 56 out of 104 patients) due to the tissue coming from the metastatic site or there was insufficient material to perform IHC analysis. Data on anemia and iron status between patients with CKD and preserved kidney function may be unreliable since only 16 patients of the 103 total were identified with CKD. In this regard, despite not being significant, the decreased hemoglobin and hematocrit trend in the CKD group may be due to insufficient statistical power. Additionally, we did not collect data on other comorbidities and drug usage that might affect hemoglobin level and iron status. Of note, inflammation can affect iron status, and markers of inflammation were not assessed in our study (e.g., C-reactive protein (CRP)); nevertheless, elevated ferritin levels in the whole cohort already reflect inflammation in these patients. Our study has several strengths: We analyzed micronutrient deficiencies in patients with PDAC initiating systemic chemotherapy that provided reliable information on anemia’s causes in this group of patients, excluding the myelosuppressive effect of chemotherapy. We also checked how renal function affects anemia in PDAC patients. We performed ZIP14 staining on PDAC tissue, which has not been conducted before on PDAC tissue.

## 5. Conclusions

Anemia is a fundamental problem in PDAC patients and occurs in 42%, whereas ID is noted in half before the onset of systemic treatment. Vitamin B12 and folate deficiency anemia are rare and occur in less than 5% of patients. More data are needed to evaluate the prevalence of folate and vitamin B12 deficiency in other cancers and the benefit of supplementing these micronutrients on anemia and patients’ quality of life. Chronic kidney diseases did not affect the development of anemia in PDAC patients. Additionally, our study showed that HEP expression on an autocrine level in PDAC is of minor importance. Interestingly, ZIP14 expression differs between more advanced tumors, with a stronger expression in smaller tumors, indicating the role of ZIP14 as an NTBI or other metal transporter in PDAC. The mass of mitochondria correlated with ZIP14 in PDAC tissue, indicating ZIP14 as possible transporter for ions necessary for mitochondria’s functioning—and potentially participating in mitochondria’s metabolism. Iron deposits were not detected in the PDAC tissue in the Perl’s Prussian blue staining outside macrophages, but the GEPIA database analysis; with elevated TfR1, FTH, FTL expressions in PDAC compared to normal pancreas; provided information about potential high iron usage within PDAC cells. 

## Figures and Tables

**Table 1:** Clinical data and laboratory findings in patients with PDAC.

Parameter	Adjuvant (*n* = 36)	Palliative (*n* = 67)	Total (*n* = 103)
Age, year ± SD	65.6 ± 8.5	65.8 ± 8.0	66.1 ± 8.2
Sex female/male, *n* (%)	20 (55.6)/16 (44.4)	45 (67.2)/22 (32.8)	65 (63.1)/38 (36.9)
Histopathological type (tub/por/muc/sq) *n*	31/1/2/1	19/1/0/1	50/2/2/2
Location (Ph/Pb/Pt) *n*	29/4/2	16/4/1	45/8/3
pT, 1/2/3/4 * *n*	2/21/12/0	n/a	n/a
pN, 1/2/3 * *n*	11/20/4	n/a	n/a
Stage (IA/IB/IIA/IIB/III/IV) * *n*	1/8/3/18/4/1	IV-21	1/8/3/18/4/22
Vascular invasion, no/yes *n*	11/24	n/a	n/a
Neural invasion, no/yes *n*	3/32	n/a	n/a
Grade, 1/2/3 *n*	2/28/5	0/20/1	2/48/6
Resection, R 0/1/2/ *n*	18/14/3	n/a	n/a
Material from surgery/biopsy *n*	35/0	14/7	49/7
Cht regimen, gem/FOLFIRINOX/gem + npxl/ *n*	32/3/0	17/0/4	49/3/4
Adjuvant, cht failure/complete *n*	13/22	n/a	n/a
Anemia, *n* (%)	15 (41.6)	29 (43.2)	44 (42.7)
Mild anemia, *n* (%)	10 (27.8)	23 (34.3)	33 (32.0)
Moderate anemia, *n* (%)	5 (13.8)	6 (8.9)	11 (10.7)
Hb (g/dL)	12.1 ± 1.7	12.2 ± 1.4	12.0 ± 1.5
Ht (%)	36.7 ± 4.4	36.6 ± 4.2	36.6 ± 4.0
MCV (fL)	89.7 ± 4.9	89.8 ± 5.4	89.7 ± 5.2
MCH (pg)	29.3 ± 2.1	29.9 ± 2.1	29.7 ± 2.1
MCHC (g/dL)	32.3 ± 2.1	33.2 ± 0.8	32.9 ± 1.4
Microcytic anemia, *n* (%)	1 (2.8)	6 (8.9)	7 (6.7)
Macrocytic anemia, *n* (%)	1 (2.8)	1 (1.5)	2 (1.8)
Hypochromic anemia, *n* (%)	2 (5.5%)	0	2 (1.8)
ID, n (%)	16 (44.4)	37 (55.2)	53 (51.4)
Functional ID, *n* (%)	9 (25.0)	28 (41.8)	37 (35.9)
Functional ID with anemia, *n* (%)	5 (13.9)	18 (26.9)	23 (22.3)
Absolute ID, *n* (%)	7 (19.4)	9 (13.4)	16 (15.5)
Absolute ID with anemia, *n* (%)	5 (13.9)	3 (4.5)	8 (7.8)
TIBC (µg/dL)	299.4 ± 69.6	279.5 ± 62.8	286.2 ± 70.0
Serum iron (µg/dL)	60.4 ± 27.2	58.3 ± 37.6	59.0 ± 34.4
Transferrin (mg/dL)	233.2 ± 46.3	225.5 ± 62.8	228.1 + 57.6
Ferritin (ng/mL)	302.3 ± 300.8	326.3 ± 274.0	318.3 ± 281.8
TSAT (%)	21.5 ± 11.3	21.0 ± 13.4	21.1 ± 12.7
Vit. B12 concentration (pg/mL)	649.6 ± 505.6	716.3 ± 479.6	693.6 ± 486.9
Vit. B12 deficiency, *n* (%)	3 (8.3)	2 (2.9)	5 (4.8)
Vit. B12 deficiency with anemia, *n* (%)	2 (5.5%)	0	2 (1.8)
Vit. B12 >800 pg/mL, *n* (%)	7 (19.4)	18 (26.8)	25 (24.3)
Folate concentration (ng/mL) *	9.4 ± 5.1	7.3 ± 3.2	8.0 ± 4.0
Folate deficiency, *n* (%)	1 (2.8)	7 (10.4)	8 (7.7)
Folate deficiency and anemia, *n* (%)	0	3 (4.5)	3 (2.9)
Creatinine concentration (mg/dL)	0.7 ± 0.1	0.7 ± 0.2	0.7 ± 0.2
GFR (mL/min/1.73 m^2^)	82.3 ± 18.9	80.4 ± 22.5	81.1 ± 21.6
CKD, *n (%)*	5 (13.9)	11 (16.4)	16 (15.5)
CKD and anemia, *n* (%)	4 (11.1)	5 (7.5)	9 (8.7)

Tub, tubular adenocarcinoma; por, poorly differentiated adenocarcinoma; muc, mucinous adenocarcinoma; sq, adenosquamous carcinoma; Ph, pancreas head; Pb, pancreas body; Pt, pancreas tail; * TNM according to UICC 8th edition; pT, tumor; pN, lymph nodes; R0, complete resection; R1, microscopic residual tumor; R2 macroscopic residual tumor; cht, chemotherapy regimen; gem, gemcitabine in monotherapy; gem + npxl, gemcitabine with nab-paclitaxel; n/a, not applicable. Anemia, Hb < 12 g/dL; mild anemia, 11.9–10.0 g/dL; moderate anemia 9.9–8.0 g/dL; ID, iron deficiency (TSAT < 20%); absolute iron deficiency (TSAT < 20%; SF < 100 ng/mL); functional iron deficiency (TSAT < 20; SF > 100 ng/mL); Hb, hemoglobin level, (normal range, 12–18 g/dL); Ht, hematocrit (36–48%); MCV, mean cellular volume (80–100 fL); MCH, mean corpuscular hemoglobin (27–33 pg); MCHC, mean corpuscular hemoglobin concentration (31–36 g/dL); microcytic anemia (MCV < 80 fL); macrocytic anemia (MCV >100 fL); hypochromic anemia (MCHC < 31 g/dL); TIBC, total iron-binding capacity (149–504 µg/dL); serum iron (37–158 µg/dL); transferrin (250–320 mg/dL); ferritin, serum ferritin (30–400 ng/mL); TSAT, transferrin saturation (20–30%); vit. B12, vitamin B12 (200–800 pg/mL); folate concentration (4–26.8 ng/mL); folate deficiency (<4 ng/mL); creatinine concentration (0.6 mg/dL–1.0 mg/dL); GFR, glomerular filtration rate, calculated with Cockcroft–Gault formula (mL/min/1.73 m^2^); CKD, chronic kidney disease (GFR < 60 mL/min/m 1.73 m^2^). * *p* value = 0.01. Test used for the statistical analysis: Mann-U-Whitney, and a *p*-value < 0.05 was adopted as the significance level. Values are displayed as mean ± SD or *n* (%) unless stated otherwise.

**Table 2:** Impact of chronic kidney disease on anemia and serum-iron-related parameters in patients with PDAC.

Parameter	Chronic Kidney Disease (*n* = 16)	Preserved Kidney Function (*n* = 87)	*p*-Value
Hb (g/dL)	11.5 ± 1.2	12.3 ± 1.6	0.07
Ht (%)	35.1 ± 3.5	36.0 ±4.3	0.07
MCV (fL)	93.6 ± 5.3	89.0 ± 4.9	0.002
MCH (pg)	30.8 ± 1.8	29.5 ± 2.1	
MCHC (g/dL)	32.8 ± 1.1	32.9 ± 1.5	
TIBC (µg/dL)	269.1 ± 65.4	289.5 ± 70.8	
Serum iron (µg/dl)	63.4 ± 24.5	58.1 ± 36.1	
Transferrin (mg/dl)	215.6 ± 57.4	230.5 ± 57.7	
Ferritin (ng/mL)	351.8 ± 324.0	311. 6 ± 274.5	
TSAT (%)	23.5 ± 10.9	20.7 ± 13.0	

Hb, hemoglobin level (normal range 12–18 g/dL); Ht, hematocrit (36–48%); MCV, mean cellular volume (80–100 fL); MCH, mean corpuscular hemoglobin (27–33 pg); MCHC, mean corpuscular hemoglobin concentration (31–36 g/dL); TIBC, total iron binding capacity (149–504 µg/dL); serum iron (37–158 µg/dL); transferrin (250–320 mg/dL); ferritin, serum ferritin (30–400 ng/mL); TSAT, transferrin saturation (20–30%); chronic kidney disease (GFR < 60 mL/min/m 1.73 m^2^); GFR, glomerular filtration rate, calculated with Cockcroft–Gault formula. Test used for the statistical analysis: Mann-U-Whitney, and a *p*-value < 0.05 was adopted as the significance level.

## Data Availability

The data presented in this study are available upon request from the corresponding author. The data are not publicly available due to protection of patients’ privacy.

## Abbreviations

CKD: Chronic kidney disease. FPN: ferroportin. FTH: ferritin heavy chain. FTL: ferritin light chain. GFR: glomerular filtration rate. HEP: hepcidin. Hb: hemoglobin concentration. Ht: hematocrit concentration. ID: iron deficiency. IHC: immunohistochemical. MIT: mitochondrial mass. NTBI: non-transferrin bound iron, PDAC: pancreatic ductal adenocarcinoma. SF: serum ferritin. TSAT: transferrin saturation. TfR1: transferrin 1 receptor. ZIP: Zrt- and Irt-like protein.

## References

[1] Ludwig H., Van Belle S., Barrett-Lee P., Birgegård G., Bokemeyer C., Gascón P., Kosmidis P., Krzakowski M., Nortier J., Olmi P. (2004). The European Cancer Anaemia Survey (ECAS): A large, multinational, prospective survey defining the prevalence, incidence, and treatment of anaemia in cancer patients. Eur. J. Cancer.

[2] Ludwig H., Müldür E., Endler G., Hübl W. (2013). Prevalence of iron deficiency across different tumors and its association with poor performance status, disease status and anemia. Ann. Oncol..

[3] Adamson J.W. (2008). The Anemia of Inflammation/Malignancy: Mechanisms and Management. Hematol. Am. Soc. Hematol. Educ. Program.

[4] Busti F., Marchi G., Ugolini S., Castagna A., Girelli D. (2018). Anemia and Iron Deficiency in Cancer Patients: Role of Iron Replacement Therapy. Pharmaceuticals.

[5] Thavarajah S., Choi M.J. (2019). The Use of Erythropoiesis-Stimulating Agents in Patients with CKD and Cancer: A Clinical Approach. Am. J. Kidney Dis..

[6] Van Eeden R., Rapoport B.L. (2016). Current trends in the management of anaemia in solid tumours and haematological malignancies. Curr. Opin. Support. Palliat. Care.

[7] Bastit L., Vandebroek A., Altintas S., Gaede B., Pintér T., Suto T.S., Mossman T.W., Smith K.E., Vansteenkiste J.F. (2008). Randomized, Multicenter, Controlled Trial Comparing the Efficacy and Safety of Darbepoetin Alfa Administered Every 3 Weeks with or Without Intravenous Iron in Patients with Chemotherapy-Induced Anemia. J. Clin. Oncol..

[8] Latenstein A.E.J., van Gerven R., Grevers F., Pek C.J., Koerkamp B.G., Hartog H., A E de van der Schueren M., Besselink M.G., van Eijck C.H.J. (2021). Micronutrient deficiencies and anaemia in patients after pancreatoduodenectomy. Br. J. Surg..

[9] Jackson T., Vedantam S., Bradshaw R., Cho E., Lim J., Nagatomo K., Osman H., Jeyarajah D.R. (2020). Unrecognized anemia after Whipple—Should we learn from gastric bypass?. Expert Rev. Gastroenterol. Hepatol..

[10] Armstrong T., Strommer L., Ruiz-Jasbon F., Shek F., Harris S., Permert J., Johnson C. (2007). Pancreaticoduodenectomy for Peri-Ampullary Neoplasia Leads to Specific Micronutrient Deficiencies. Pancreatology..

[11] Wang Y., Yu L., Ding J., Chen Y. (2019). Iron Metabolism in Cancer. Int. J. Mol. Sci..

[12] Porporato P.E., Filigheddu N., Pedro J.M.B.-S., Kroemer G., Galluzzi L. (2018). Mitochondrial metabolism and cancer. Cell Res..

[13] Brown R.A.M., Richardson K.L., Kabir T.D., Trinder D., Ganss R., Leedman P.J. (2020). Altered Iron Metabolism and Impact in Cancer Biology, Metastasis, and Immunology. Front. Oncol..

[14] Jeong S.M., Hwang S., Seong R.H. (2016). Transferrin receptor regulates pancreatic cancer growth by modulating mitochondrial respiration and ROS generation. Biochem. Biophys. Res. Commun..

[15] Reyes-Castellanos G., Masoud R., Carrier A. (2020). Mitochondrial Metabolism in PDAC: From Better Knowledge to New Targeting Strategies. Biomedicines.

[16] Muckenthaler M.U., Rivella S., Hentze M.W., Galy B. (2017). A Red Carpet for Iron Metabolism. Cell.

[17] Pasricha S.-R., Tye-Din J., Muckenthaler M.U., Swinkels D.W. (2021). Iron deficiency. Lancet.

[18] Arezes J., Jung G., Gabayan V., Valore E., Ruchala P., Gulig P.A., Ganz T., Nemeth E., Bulut Y. (2015). Hepcidin-Induced Hypoferremia Is a Critical Host Defense Mechanism against the Siderophilic Bacterium Vibrio vulnificus. Cell Host Microbe.

[19] Aydemir T.B., Cousins R.J. (2018). The Multiple Faces of the Metal Transporter ZIP14 (SLC39A14). J. Nutr..

[20] Mertens C., Mora J., Ören B., Grein S., Winslow S., Scholich K., Weigert A., Malmström P., Forsare C., Fernö M. (2018). Macrophage-derived lipocalin-2 transports iron in the tumor microenvironment. Oncoimmunology.

[21] Aapro M., Beguin Y., Bokemeyer C., Dicato M., Gascón P., Glaspy J., Hofmann A., Link H., Littlewood T., Ludwig H. (2018). Management of anaemia and iron deficiency in patients with cancer: ESMO Clinical Practice Guidelines. Ann. Oncol..

[22] Wish J.B. (2006). Assessing iron status: Beyond serum ferritin and transferrin saturation. Clin. J. Am. Soc. Nephrol..

[23] Brierley J.D., Gospodarowicz M.K., Union for International Cancer Control (2016). TNM Classification of Malignant Tumors.

[24] Tang Z., Li C., Kang B., Gao G., Li C., Zhang Z. (2017). GEPIA: A web server for cancer and normal gene expression profiling and interactive analyses. Nucleic Acids Res..

[25] Gilreath J.A., Stenehjem D.D., Rodgers G.M. (2014). Diagnosis and treatment of cancer-related anemia. Am. J. Hematol..

[26] Madeddu C., Gramignano G., Astara G., Demontis R., Sanna E., Atzeni V., Macciò A. (2018). Pathogenesis and Treatment Options of Cancer Related Anemia: Perspective for a Targeted Mechanism-Based Approach. Front. Physiol..

[27] Kelly L., White S., Stone P. (2007). The B12/CRP index as a simple prognostic indicator in patients with advanced cancer: A confirmatory study. Ann. Oncol..

[28] Obeid R. (2022). High Plasma Vitamin B12 and Cancer in Human Studies: A Scoping Review to Judge Causality and Alternative Explanations. Nutrients.

[29] Ueda N., Takasawa K. (2018). Impact of Inflammation on Ferritin, Hepcidin and the Management of Iron Deficiency Anemia in Chronic Kidney Disease. Nutrients.

[30] Hamley S. (2017). The effect of replacing saturated fat with mostly n-6 polyunsaturated fat on coronary heart disease: A meta-analysis of randomised controlled trials. Nutr. J..

[31] Ciniselli C.M., De Bortoli M., Taverna E., Varinelli L., Pizzamiglio S., Veneroni S., Bonini C., Orlandi R., Verderio P., Bongarzone I. (2015). Plasma hepcidin in early-stage breast cancer patients: No relationship with interleukin-6, erythropoietin and erythroferrone. Expert Rev. Proteom..

[32] Pinnix Z.K., Miller L.D., Wang W., D’agostino R., Kute T., Willingham M.C., Hatcher H., Tesfay L., Sui G., Di X. (2010). Ferroportin and Iron Regulation in Breast Cancer Progression and Prognosis. Sci. Transl. Med..

[33] Tesfay L., Clausen K.A., Kim J.W., Hegde P., Wang X., Miller L.D., Deng Z., Blanchette N., Arvedson T., Miranti C.K. (2015). Hepcidin Regulation in Prostate and Its Disruption in Prostate Cancer. Cancer Res.

[34] Jung M., Mertens C., Tomat E., Brüne B. (2019). Iron as a Central Player and Promising Target in Cancer Progression. Int. J. Mol. Sci..

[35] Toshiyama R., Konno M., Eguchi H., Asai A., Noda T., Koseki J., Asukai K., Ohashi T., Matsushita K., Iwagami Y. (2018). Association of iron metabolic enzyme hepcidin expression levels with the prognosis of patients with pancreatic cancer. Oncol. Lett..

[36] Xu X.M., Wang C.G., Zhu Y.D., Chen W.H., Shao S.L., Jiang F.N., Liao Q.D. (2016). Decreased expression of SLC39A14 is associated with tumor aggressiveness and biochemical recurrence of human prostate cancer. Onco Targets Ther..

[37] Zhu B., Huo R., Zhi Q., Zhan M., Chen X., Hua Z.-C. (2021). Increased expression of zinc transporter ZIP4, ZIP11, ZnT1, and ZnT6 predicts poor prognosis in pancreatic cancer. J. Trace Elements Med. Biol..

[38] Shakri A.R., Zhong T.J., Ma W., Coker C., Kim S., Calluori S., Scholze H., Szabolcs M., Caffrey T., Grandgenett P.M. (2020). Upregulation of ZIP14 and Altered Zinc Homeostasis in Muscles in Pancreatic Cancer Cachexia. Cancers.

[39] Aydemir T.B., Troche C., Kim M.-H., Cousins R.J. (2016). Hepatic ZIP14-mediated Zinc Transport Contributes to Endosomal Insulin Receptor Trafficking and Glucose Metabolism. J. Biol. Chem..

[40] Daniels T.R., Delgado T., Rodriguez J.A., Helguera G., Penichet M.L. (2006). The transferrin receptor part I: Biology and targeting with cytotoxic antibodies for the treatment of cancer. Clin. Immunol..

[41] Daniels T.R., Delgado T., Helguera G., Penichet M.L. (2006). The transferrin receptor part II: Targeted delivery of therapeutic agents into cancer cells. Clin. Immunol..

[42] Schonberg D.L., Miller T.E., Wu Q., Flavahan W.A., Das N.K., Hale J.S., Hubert C.G., Mack S.C., Jarrar A.M., Karl R.T. (2015). Preferential Iron Trafficking Characterizes Glioblastoma Stem-like Cells. Cancer Cell.

[43] Poh A.R., Ernst M. (2021). Tumor-Associated Macrophages in Pancreatic Ductal Adenocarcinoma: Therapeutic Opportunities and Clinical Challenges. Cancers.

[44] Zhu Y., Herndon J.M., Sojka D.K., Kim K.-W., Knolhoff B.L., Zuo C., Cullinan D.R., Luo J., Bearden A.R., Lavine K.J. (2017). Tissue-Resident Macrophages in Pancreatic Ductal Adenocarcinoma Originate from Embryonic Hematopoiesis and Promote Tumor Progression. Immunity.

